# Endosalpingiosis of urinary bladder: report on a rare entity

**DOI:** 10.1259/bjrcr.20190129

**Published:** 2020-03-10

**Authors:** Saurabh Maheshwari, Venkatraman Bhat, Karthik Gadabanahalli, Nalini Raju, Prashant Kulkarni

**Affiliations:** 1 Department of Radiology, Narayana Health, Bengaluru, India; 2 Department of Pathology, Narayana Health, Bengaluru, Karnataka, India; 3 Department of Urology, Narayana Health, Bengaluru, Karnataka, India

## Abstract

A case of endosalpingiosis of the urinary bladder is presented with imaging features on sonography and CT. Patient presented with right flank pain, dysuria and haematuria. She had h/o right renal calculus and abdominal hysterectomy 15 years ago. On sonography a polypoidal filling defect was noted and possibility of a bladder neoplasia was suggested. On cystoscopy and removal of the lesion and subsequent histo-pathological analysis revealed the diagnosis of endosalphingiosis. This report emphasizes the need for evaluation of all clinical inputs while considering the differential diagnosis of an intraluminal bladder lesion. Imaging appearance and aetio-pathology of the rare intra vesical lesion is highlighted.

## Clinical presentation

A 46-year-old female presented to the outpatient department of the hospital in with complaints of right flank pain associated with dysuria and haematuria. There was past history of abdominal hysterectomy 15 years ago. General and pelvic examinations were unremarkable. Routine urine examination revealed blood products (RBCs—6–8/hpf) and pus cells (4–6/hpf)

## Investigations/imaging findings

**Figure 1.**
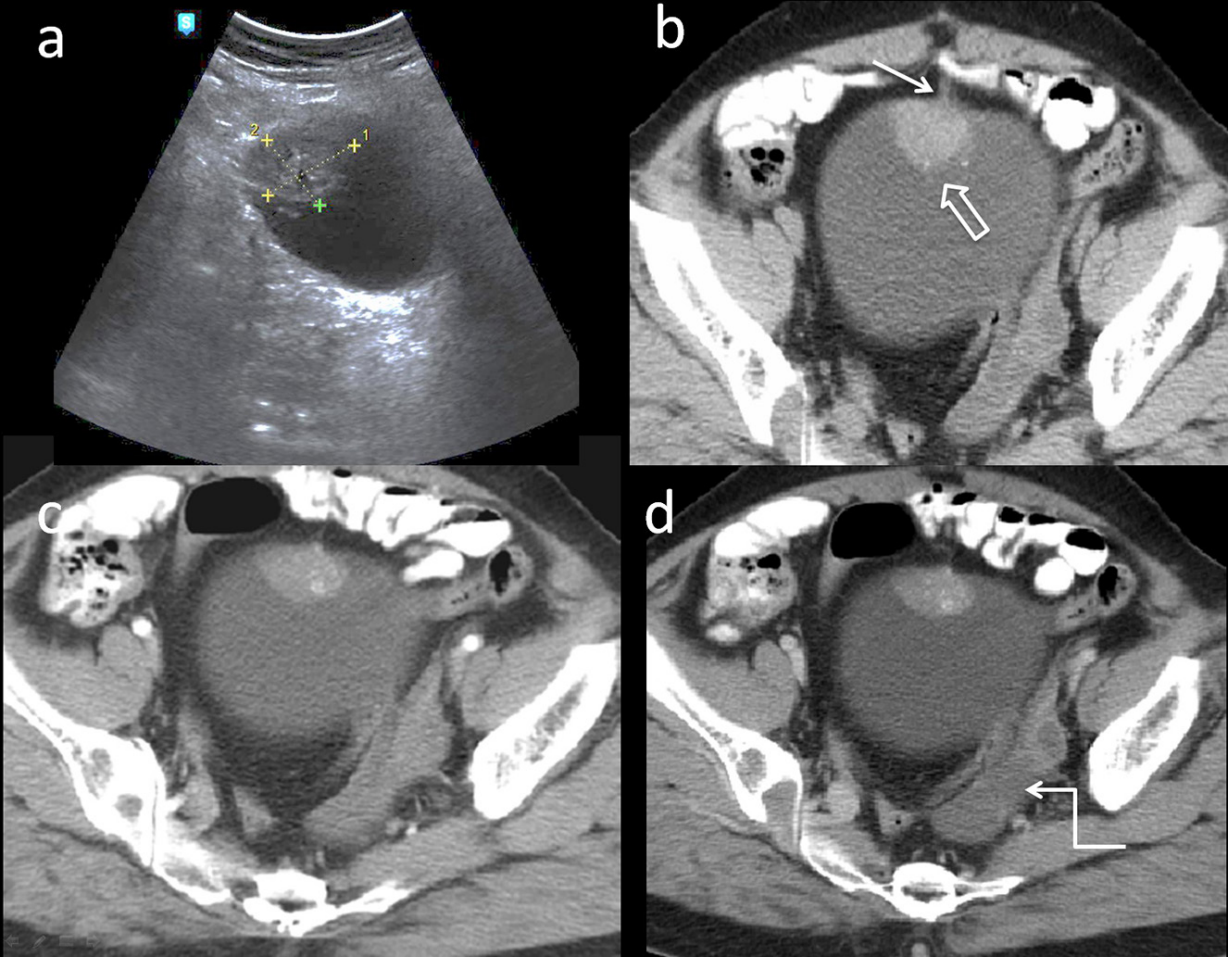
(a) 46-year-old female presented with haematuria. Sagittal ultrasound image of urinary bladder showing hypoechoic polypoidal lesion with hyperechoic foci along the anterosuperior wall of urinary bladder. Figure 1 (b) Non-contrast axial CT image of urinary bladder reveal a well-defined soft tissue density lesion with few calcific specks (open arrow) in anterosuperior wall of urinary bladder. Small, linear soft tissue density is noted between anterior abdominal wall and urinary bladder (arrow). Post-contrast axial CT images in early arterial and venous phase demonstrates heterogeneous enhancement of the lesion (c, d). Also there is left hydrosalpinx (Ziz-zag arrow).

**Figure 2.**
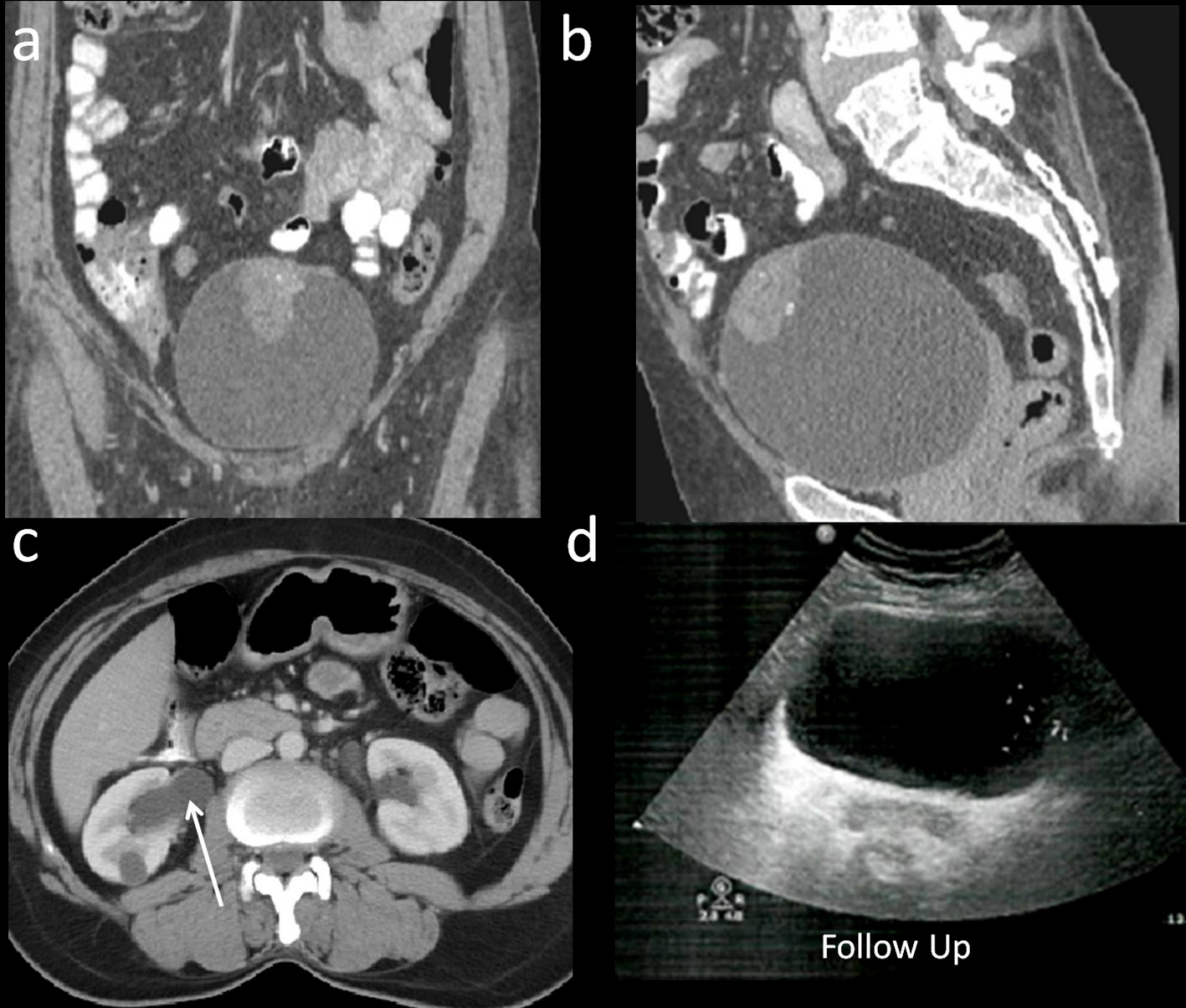
46-year-old-female with haematuria. Coronal and Sagittal reconstructions of urinary bladder in venous phase (a, b) shows filling defect in urinary bladder containing calcific foci. There is minimal thickening of bladder wall on left side. Axial image at the level of kidneys show mild dilatation of right renal pelvis (arrow). 6 month follow-up sonogram (d) of urinary bladder shows normal lumen and contours of urinary bladder.

An ultrasound examination of abdomen and pelvis showed focal hypoechoic polypoidal lesion, measuring 3.4 × 2.8 cm, along the anterosuperior wall of urinary bladder. (Figure 1a). Tiny hyperechoic foci were noted within the lesion. Outline of the adjacent urinary bladder appeared indistinct. The uterus was not seen (prior hysterectomy). There was a cystic area in left adnexa; ovaries were not visualised. CT examination performed for assessment of extent of a suspected neoplastic lesion, showed a well-defined soft tissue density lesion with few calcific specks along the antero superior wall of urinary bladder (Figure 1,b). Lesion showed heterogeneous contrast enhancement in early arterial phase persisting on to the venous phase (Figure 1c,d). Intraluminal component was merging with bladder wall with minimal thickening of urinary bladder wall on left side of the lesion (Figure 2a,b). Rest of the bladder wall was normal. There was a *in situ* calcyceal calculus and a simple renal cyst in the right kidney with mildly dilatation of renal pelvis (Figure 2c). There was no pelvic lymphadenopathy. There was left hydrosalpinx. On imaging possibilities like endometriosis, mucosal neoplastic or granulomatous lesion of urinary bladder was considered.

## Treatment/management

**Figure 3.**
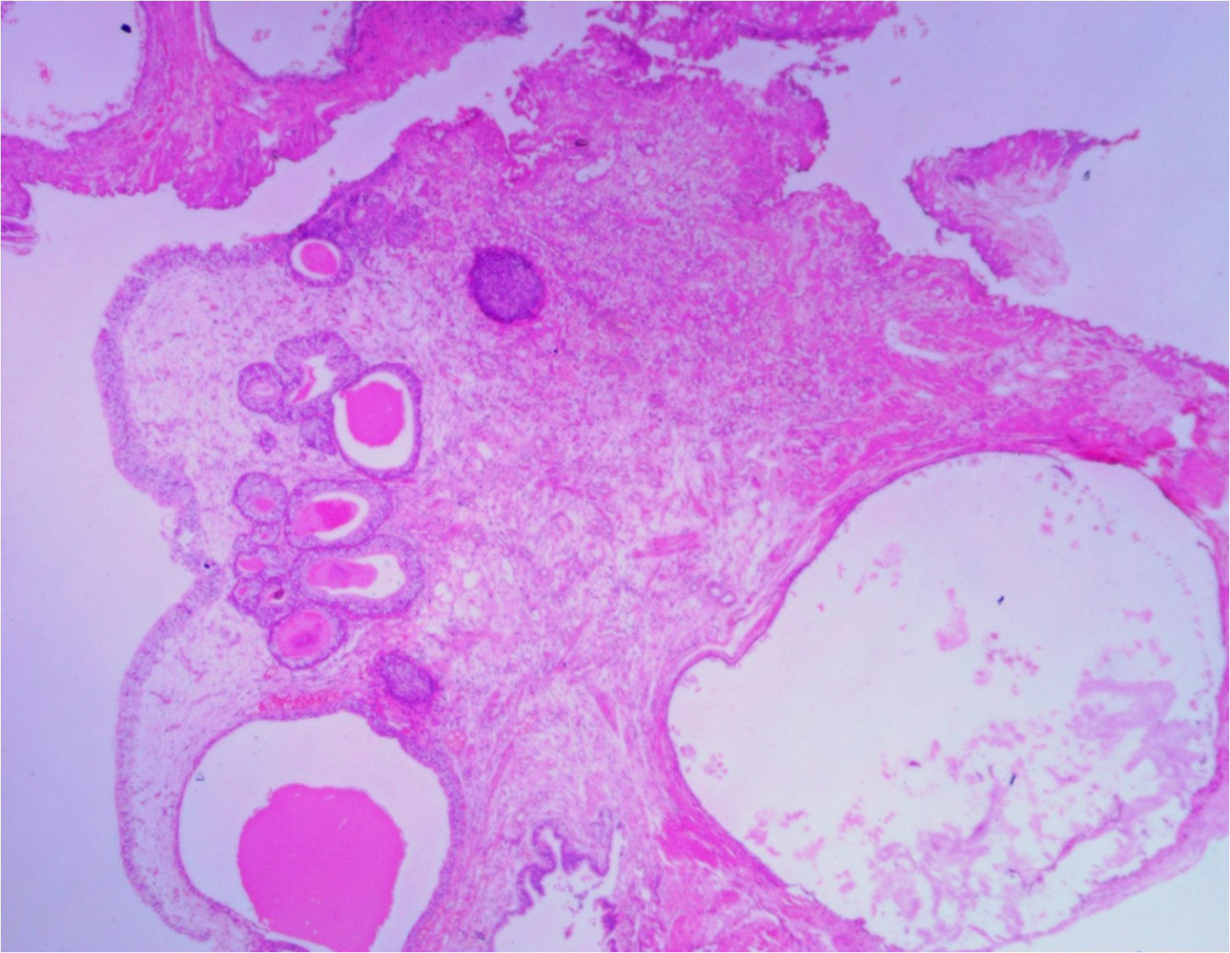
Microscopic exam with H&E stain shows normal epithelium. Lamina propria shows multiple cystic spaces of varying size lined by ciliated cuboid epithelium mimicking tubal epithelium.

Cystoscopy and transurethral resection of the lesion was performed. Gross examination of resected specimen showed a tan-brownish soft tissue aggregate. Histopathological examination revealed fragments of bladder tissue lined by predominantly benign urothelium. No dysplasia or carcinoma. The muscularis propria shows scattered variable shaped cystic glands lined by benign appearing tubal type ciliated columnar epithelium. There were no endometrial glands or stroma. Findings were suggestive of endosalpingiosis (Figure 3).

## Outcome

The patient was subsequently reviewed and followed-up after 6 month with ultrasound of abdomen and pelvis. It revealed no recurrent/residual mass lesion in urinary bladder (Figure 2d). Presently, patient is on follow-up and free of symptoms.

## Discussion

Endosalpingiosis is a rare benign condition, can occur in isolation or part of the entity of Mullarianosis. It is characterised by the presence of glands lined by ciliated tubal-type epithelium in locations other than the fallopian tubes.^1^ It is a non-neoplastic lesion which is similar to entities like endometriosis and endocervicosis. Condition was originally described by Sampson in 1930, who found epithelium resembling the fallopian tube in the ectopic locations..^2^

Endosalpingiosis of the urinary bladder, in particular, is an extremely rare condition, with five cases described in literature,^3^ characterized histopathologically by the presence of tubal type epithelium without other müllerian components in the lamina propria and muscularis propria of the urinary bladder.^2,3^ It was described as an isolated entity by Young and clement in 1996.^4,5^ Imaging appearance of the entity is scanty, hence awareness of the condition is helpful in broadening the differential diagnosis of bladder mass. We report a case which presented as a diagnostic challenge in imaging studies.

The pathogenesis of müllerianosis and endosalpingiosis remains completely unresolved. However, some hypotheses have been proposed. The implantation theory by Young and Clement proposes that müllerian tissue can implant in the urinary bladder wall during caesarean section or pelvic surgery.^4^ This condition mainly affects pre-menopausal females with an average age of 44.6 years. The patients usually present with complains of haematuria or dysuria.^2–5^

On imaging examination, findings are non-specific, patients presenting with a polypoidal intraluiminal vesical mass with a mural component, thus wide range of differential diagnosis is considered, mainly consisting of endometiosis and other lesions of neoplastic or granulomatous aetiology. There are occasional reports about the imaging appearances of the lesion in the literature, describing findings on intravenous urography and CT. In our patient, demonstration of band like extravesical soft tissue density adjacent to bladder lesion is interesting as there is an implantation theory as a possible mechanism for aetiology of endosalpingiosis. However, a collective analysis of large number of cases is needed to establish such a connection. Also, presence of intralesional calcific specks makes the observation unique. Pathology reports do not report this component, hence could represent secondary findings due to encrustation or salt deposition. Sonography generally identifies the lesion, although information regarding adjacent region around the bladder is suboptimal. Other modalities like CT, possibly MRI have a role in elucidating special features and the total extent of the abnormality. In one previously described case,^5^ lesions appears T2 hypointense, hence MRI examination may have a role in differentiating from inflammatory and some neoplastic lesions which are T2 hyperintense. Cystoscopic examination is the common next step in management either to obtain a tissue sample or implement a treatment process. It may reveal a vascular lesion or mucosal covered polyp. Specific diagnosis is not possible without tissue analysis.

The histopathological diagnosis is mandatory for the diagnosis of müllerianosis and endosalpingiosis of the urinary bladder. Though the diagnosis is obvious on histopathology, there are few mimics to be differentiated such as cystitis glandularis, urachal remnant, nephrogenic adenoma, and adenocarcinoma.^6^

## Teaching point

1. In the differential diagnosis of intravesical filling defect in a female patient with prior hysterectomy should include unusual causes like endometrioses or endosalphngiosis
2. Imaging appearance of endosalphingiosis of urinary bladder is often non-specific, some unusual findings include extratuminal soft tissue component (T2 hypointensity on MRI examination)—hence histopathological confirmation is essential.
